# ATP-Dependent Chromatin Remodeling Complex in the Lineage Specification of Mesenchymal Stem Cells

**DOI:** 10.1155/2020/8839703

**Published:** 2020-09-09

**Authors:** Wen Du, Daimo Guo, Wei Du

**Affiliations:** ^1^State Key Laboratory of Oral Diseases, National Clinical Research Center for Oral Diseases, Department of Prosthodontics, West China Hospital of Stomatology, Sichuan University, Chengdu 610041, China; ^2^State Key Laboratory of Oral Diseases, National Clinical Research Center for Oral Diseases, West China Hospital of Stomatology, Sichuan University, Chengdu 610041, China; ^3^State Key Laboratory of Oral Diseases, National Clinical Research Center for Oral Diseases, Department of Cariology and Endodontics, West China Hospital of Stomatology, Sichuan University, Chengdu 610041, China

## Abstract

Mesenchymal stem cells (MSCs) present in multiple tissues can self-renew and differentiate into multiple lineages including the bone, cartilage, muscle, cardiac tissue, and connective tissue. Key events, including cell proliferation, lineage commitment, and MSC differentiation, are ensured by precise gene expression regulation. ATP-dependent chromatin alteration is one form of epigenetic modifications that can regulate the transcriptional level of specific genes by utilizing the energy from ATP hydrolysis to reorganize chromatin structure. ATP-dependent chromatin remodeling complexes consist of a variety of subunits that together perform multiple functions in self-renewal and lineage specification. This review highlights the important role of ATP-dependent chromatin remodeling complexes and their different subunits in modulating MSC fate determination and discusses the proposed mechanisms by which ATP-dependent chromatin remodelers function.

## 1. Introduction

Mesenchymal stem cells (MSCs), also known as mesenchymal stromal cells, are multipotent stromal cells that can differentiate into a variety of mesoderm cell types, including osteoblasts, chondrocytes, myocytes, and adipocytes [1–3]. MSCs are a heterogeneous subset of stem cells that can be obtained from different locations of adult tissues including the bone marrow, adipose tissue, and other sources [4–6]. Studies have also indicated that MSCs can differentiate into endoderm and ectoderm lineages, including hepatocytes, epidermal-like cells, neurons, and other cell fates [7–10]. MSCs are a great choice for tissue engineering, regenerative medicine, and clinical therapy. The MSC differentiation process is regulated by different regulatory mechanisms like signaling molecules and epigenetic modifications [11, 12]. All regulatory mechanisms determine the selective transcription of genes with discrete combinations. This selective transcription will define the differentiation process and subsequently determine the specific lineage. Knowledge of how specific lineage differentiation occurs and how epigenetic modifications are involved in this process will accelerate the research and development of cell-based tissue engineering therapy. The current review summarizes our understanding of how ATP-dependent chromatin remodeling complexes regulate multilineage MSC differentiation.

## 2. ATP-Dependent Chromatin Remodeling Complexes

Specific gene expression programs, which depend largely on the organization of the associated chromatin, define a variety of cellular processes like differentiation, proliferation, and stemness [13]. ATP-dependent chromatin alterations, as one of the major factors that affect chromatin state, can determine a specific gene's transcription level [14–16]. ATP-dependent chromatin alteration is achieved by multisubunit ATP-dependent chromatin remodeling complexes. These complexes can utilize ATP hydrolysis-derived energy to remodel nucleosome structure, thus modulating transcription factor binding to cognate DNA.

ATP-dependent chromatin remodeling complexes mainly consist of an ATPase and multiple subunits. The ATPase subunit hydrolyzes ATP, while the associated subunits regulate ATPase catalytic activity and genome binding. Therefore, different combinations of ATPase and associated subunits result in various chromatin remodeling complexes with different functions [17, 18]. All ATP-dependent chromatin remodeling complexes include an ATPase subunit from the SNF2 family, which can be divided into four different subfamilies, including SWI/SNF (switch/sucrose nonfermentable), ISWI (imitation SWI), CHD (chromodomain helicase DNA-binding), and INO80 (SWI2/SNF2 related (SWR)) based on sequence similarity between their ATPase domains [16, 19, 20]. The diversity of different isoforms of associated subunits defines a variety of particular properties that are suited to the specific tissue type and assist in recruiting the complexes to specific genomic loci. Complexes with various subunit combinations have been detected in different cell or tissue types during development. For instance, the SWI/SNF complex with BAF60C of the BAF60 subunit functions in gene transcription in the muscles and the heart, while the BAF60A isoform of the complex has a limited role in these tissues [21, 22]. The increasingly identified tissue-specific subunits of ATP-dependent chromatin remodeling complexes indicate the necessity to better understand remodeler subunit composition and how they modulate tissue-specific gene transcription.

The interaction of ATP-dependent chromatin remodeling complexes and histone acetyltransferases (HATs) in gene transcription has been demonstrated. For example, the yeast Spt-Ada-Gcn5-acetyltransferase (SAGA) complex cooperates with the SWI/SNF complex via the cell wall integrity pathway for mandatory nucleosome displacement, which is essential for full gene expression [23]. It has also been found that the yeast and mammalian SWI/SNF complex is involved in the Rb/E2F pathway, which recruits SWI/SNF, histone deacetylases (HDACs), and histone methyltransferases (HMTs) to the E2F promoter that actively represses transcription [24]. In addition, SWI/SNF functions in both transcriptional activation and repression of the pS2 promoter via ligand-specific collaboration with HDAC1, P300, and prohibitin recruitment [25].

ATP-dependent chromatin remodeling complex can also cooperate with DNA methylation in various cellular processes. Several ATP-dependent chromatin remodeling enzymes, including the mammalian SNF2 family and ATPases ATRX and LSH (HELLS), are involved in DNA methylation at the fifth carbon of cytosine (5mC), which is an abundant epigenetic modification in vertebrate genomes [26–28]. The ATRX gene mutation, which resides on the X chromosome, causes a decrease of 30–60% in alpha globin gene expression, and this may result in an unusual form of thalassemia [27]. Patients with ATRX syndrome exhibit both hypermethylation and hypomethylation in highly repetitive elements, including satellite DNA, although the total 5mC level in the genome seems unchanged. However, a dramatic decrease (50%) in 5mC levels was present in LSH-null mice [28]. Fibroblasts from *Lsh^−/−^* mouse embryos, which lack DNA methylation from transposons, centromeric repeats, and several gene promoters, can reestablish DNA methylation and silence the misregulated genes with LSH reexpression [29]. The interactions between the complexes and other epigenetic regulation factors in different cell types provide a hint for investigating the potential role of ATP-dependent chromatin remodeling in multilineage MSC differentiation.

## 3. Role of ATP-Dependent Chromatin Remodeling in MSC Lineage Differentiation

### 3.1. SWI/SNF

The switch/sucrose nonfermentable (SWI/SNF) complex (also known as BAF) is composed of at least 15 different subunits that invariably include a core ATPase of either Brm (Brahma) or Brg1 (Brahma-related gene 1) that can provide the necessary energy to the complex for nucleosome remodeling activity [16]. BRM and BRG1 share 75% of amino acid sequences and have similar domains, including the ATPase domain, HSA domain (DNA binding), QLQ domain (protein-protein interaction), and bromodomain (acetyl-lysine histone mark recognition) [30]. Several common members including BAF155, BAF45A/B/C/D, BAF47, BAF53A/B, BAF57, BAF60A/B/C, and *β*-actin are shared by the complexes [31]. The BAF complexes can be divided into BAF250A-containing BAF-A complexes or BAF250B-containing BAF-B complexes depending on the combination of ATPase and associated subunits. Besides, BAF180- (polybromo-), BAF200-, and BRD7-containing complexes connected to the BRG1 ATPase subunit can form a polybromo-associated BAF (PBAF) complex [32].

Targeting of SWI/SNF to genomic sites is partly modulated via interactions of its associated subunits with transcription factors, histone modifications, and noncoding RNAs (ncRNAs), which were recently described. One example is the interaction between long ncRNA (lncRNA) *SChLAP1* and SMARCB1/SNF5. *SChLAP1* is an aberrantly expressed lncRNA identified in prostate cancer tissues [33], while SNF5 is a core subunit of the SWI/SNF complex that is essential for proper assembly and function of the complex [34]. Direct interaction has been found between *SChLAP1* and SNF5 in human prostate cells, while *SChLAP1* overexpression resulted in decreased SWI/SNF occupancy genome-wide [35].

SWI/SNF is critical for stem cell self-renewal and cell differentiation. BRG1, BAF47, and BAF155 depletion can impair the survival of totipotent cells and cause peri-implantation embryonic lethality in mice [36–38]. BRG1, BAF155, and BAF60A expressions are largely correlated with the reprogramming efficiency of induced pluripotent stem cells in the human population [39]. Some components of SWI/SNF have been involved in a transcriptional network that contains core transcription factors like OCT4, SOX2, and NANOG and maintains pluripotency in stem cells, and the Polycomb group (PcG) proteins modify chromatin to arrest differentiation [13, 40]. Deregulation of BRG1 expression induces MSC senescence with suppressed NANOG, and this is a part of the transcriptional circuitry that manages stem cell functions [41, 42]. BRG1 downregulation leads to an increase in DNA methyltransferase 1 (DNMT1) and Rb recruitment at the NANOG promoter, thus increasing methylation and transcriptionally silencing NANOG. *BRG1* overexpression induces BRG1 occupancy at the NANOG promoter, thereby increasing chromatin compaction and recruiting HDACs [43]. Furthermore, BRG1 knockdown in hematopoietic stem cells and progenitors was shown to result in a compromised capacity of self-renewal both *in vitro* and *in vivo* [44].

Several studies have indicated that SWI/SNF is required for osteogenic induction (Table 1). Brg1 expression was detected in *ex vivo* osteoblast cultures and in skeletal tissues of mouse embryos [45]. This expression depends on the Runx2 expression induced by BMP2. The osteocalcin (OC) promoter region can recruit BRG1 via the transcription factor C/EBP*β*; thus, BRG1 can induce OC expression regulated by RNA polymerase II [46]. Brg1 and P300 can also be recruited by Osterix (Osx) to its target gene promoter *in vivo* enhanced by p38 to form a complex that is transcriptionally active [47]. In addition, Brg1 and Baf47 can interact with C/EBP*β*-LAP^∗^, which can bind to the Ric-8B promoter. This leads to Ric-8B expression downregulation in differentiating osteoblasts [48]. Several long-term osteogenic signals specifically upregulate the PBAF subunits BAF180, BAF200, and BRD7 in MSCs. The loss of *Baf180/Baf200/Brd7* largely compromised the osteogenesis and osteolineage gene expression, while *Baf180* loss was found to impair MSC ossification *in vivo* [49]. By comprehensive mapping, SWI/SNF complexes have also been identified in cartilage-expressed transcripts [50]. FGF receptor 3 (FGFR3) expression, which is critical for developing cartilage, can be induced by BMP2. This process is mediated by Sp1, a downstream mediator, and BRG1 can induce FGFR3 expression by selectively remodeling the Sp1 binding site-containing chromatin region that is located at the FGFR3 transcription start site [51].

ATP-dependent chromatin remodeling is also essential in promoter activation during adipogenic differentiation of MSCs. BRG1 and hBRM can cooperate with C/*EΒ*P*α*, C/*EΒ*P*β*, C/EBP*δ*, and PP*Α*R*γ*2 to induce uncommitted fibroblasts into adipocytes [52, 53]. In 3T3-L1 preadipocytes and human MSCs, the depletion of BAF47 repressed adipogenic differentiation by interacting with PPAR*γ*2 and C/EBP*β* [54]. CARM1 or PRMT5, which are protein arginine methyltransferases, have also been found to mediate BRG1 binding to the PPAR*γ* promoter [55–57]. In MSC cultures with the induction of adipocyte differentiation, BRG1 overexpression promoted the mature phenotypes that were connected with an obvious increase in the expression of the differentiation markers PPAR*γ* and LPL [41]. Moreover, BRM plays an important role in maintaining the balance of MSC lineage selection between adipocytes and osteoblasts. For example, the depletion of BRM in MSCs favored the osteoblast lineage over the adipocyte lineage because BRM deletion in mice exhibited a rescued phenotype in age-related osteoporosis [58]. Furthermore, differentiated adipocytes have been found to exhibit increased miR-143 expression, while the application of antimiR-143 oligonucleotides could suppress differentiation [59]. miRNA378 expression is also relevant to adipocyte differentiation, and miRNA378 overexpression results in triglyceride accumulation and activation of lipogenic genes like PPAR*γ*2 and GLUT4 [60]. This indicated the possibility that SWI/SNF cooperates with miRNAs to participate in adipogenic differentiation.

SWI/SNF is also important for hepatocyte differentiation. During early liver development, BRM or BRG1 can decrease the expression of tryptophan oxygenase, a gene specific to the late stage [61]. During hepatocyte differentiation, the BRM expression is upregulated by degrees while BRG1 is gradually decreased. BRM or BRG1 deficiency causes decreased albumin expression in hepatocytes because BRM and BRG1 can bind to the promoter region of the albumin gene and C/EBP*α* and RB family proteins [62]. BAF60A can upregulate PPAR*α* target genes while stimulating *β*-oxidation of fat in hepatocytes [63]. Moreover, BAF47 deletion is accompanied by decreased levels of most genes involved in liver development [64]. On the other hand, the regeneration of the mammalian liver can be substantially improved by deleting Arid1a, a component of the SWI/SNF complexes. The loss of Arid1a leads to chromatin reprogramming that restricts promoter access by transcription factors like E2F4 and C/EBP*α*, which inhibit cell cycle reentry and enhance differentiation, respectively [65].

BRG1 is essential for regulating gene expression and the differentiation of cardiomyocytes [66]. In a mouse model, *Brg1* deletion in the developing heart results in dysregulated cardiac gene expression and severe cardiac morphogenesis anomalies. By mediating remodeling of promoter chromatin and BRG1 recruitment, BAF250A regulates the expression of *Mef2c*, *Nkx2-5*, and *Bmp10* during the differentiation of cardiac progenitor cells into beating cardiomyocytes [67]. In addition, BAF250A can interact with nucleosome remodeling and histone deacetylase (NURD), thus occupying the regulatory regions of genes associated with cardiomyocytes [68]. BAF250A is also critical in normal heart function, confirmed by BAF250A deletion in the sinoatrial node that stops Nkx2.5 repression, resulting in sick sinus diseases [69]. Moreover, BAF60C is crucial in reprogramming fibroblasts into cardiovascular precursors by interacting with other cardiac transcription factors, which indicates the important role of BAF60C in cardiac differentiation [70]. BAF60c can function together with Tbx5 and Gata4, the cardiomyocyte-specific transcription factors, to induce cardiomyocyte differentiation when Nodal/BMP signaling is suppressed [71]. Furthermore, BAF45A or BAF180 deficiency in mice results in hematopoietic system defects characterized by a decreased number of hematopoietic stem cells, impaired potential of long-term repopulating, and abnormal development of the hematopoietic lineage [72, 73].

MSCs could also differentiate into astrocytes or neurons when cultured with retinoic acid and neurotrophic factors derived from the brain [74]. Studies have shown that the deficiency of Brg1 and associated proteins resulted in neuronal disorders [75, 76]. During the differentiation of neurons, BAF53A is compromised and replaced by BAF53B, indicating the importance of SWI/SNF activity for proper neuron development [77]. Knockdown of *Baf45a* and/or *Baf53a* mediated by short hairpin RNA leads to decreased proliferation, while *Baf45a* overexpression improves neural progenitor cell mitosis. The SWI/SNF complex specific to neural progenitors regulates Notch and Shh signaling to promote proliferation and maintain the cells in the transition state from progenitors to postmitotic neurons [77]. Meanwhile, the depletion of BAF53B exhibits obvious defects in dendrite development and in memory [78]. Downregulated proliferation in neural progenitor cells with growth retardation in the cerebellum was also observed in mutations in Brg1 and some other SWI/SNF subunits in mice [79].

### 3.2. ISWI

ISWI complexes contain one of two conserved ATPase SMARCA5 (SNF2H) or SMARCA1 (SNF2L) along with two to four associated subunits [80]. The expression of *Snf2h*, which is critical for early embryonic development, is ubiquitous in various tissue types, while the *Snf2l* expression is restricted to the brain and postnatal reproductive tissues [80, 81]. Therefore, *Snf2h* loss leads to lethality, while mice with a *Snf2l* deficiency can still survive [82]. Furthermore, ISWI is required for nuclear organization and nucleosomal periodicity, and transcription factors depend upon specific remodeling pathways for proper genomic binding [83].

ISWI complexes containing either SNF2H or SNF2L are critical for ectoderm-derived lineage development [82, 84, 85]. In the nervous system, SNF2H is essential for neural progenitor proliferation, which can be partially compensated by SNF2L. Conditional *Snf2h* deletion compromises the proliferation of granule neuron progenitors and Purkinje cells with increased cell death, which leads to defects in postnatal neural maturation [84]. On the contrary, SNF2L was found to decrease the proliferation of neural progenitors to maintain the correct brain size. SNF2L also represses the expression of the transcription factor gene *Foxg1* by binding to its promoter region. Therefore, SNF2L is required to maintain the balance between proliferation and differentiation of neural progenitors during brain development. This can be confirmed by the increased proliferation and self-renewal of neural progenitors in conditional *Snf2l* mutants accompanied by increased FOXG1 expression [82].

ISWI is also essential in mesoderm-derived lineage differentiation. The nucleosome remodeling factor (NURF) complex, which includes SNF2L-containing ISWI, plays an important role in erythropoiesis [81]. On the other hand, SNF2H is essential for hematopoietic progenitor proliferation at an early stage during erythropoiesis [81]. Therefore, the complexes with SNF2H or SNF2L function differently during the early and late stages of hematopoiesis. SNF2L-containing NURF is also required in thymocyte development [86]. Bromodomain PHD finger transcription factor (BPTF), one of the NURF subunits, is critical for CD4 or CD8 single-positive cells to differentiate into mature T cells by regulating DNase I hypersensitivity and cooperating with the transcription factor SRF to mediate the binding of NURF to *Egr1*, a gene specific to thymocyte maturation [86]. *Bptf* mutants were not able to differentiate any ectoderm, endoderm, or mesoderm tissue types, suggesting the important role of BPTF in germ layer formation. In addition, *Bptf* mutants failed to form distal ventral endoderm, and the expression of SMAD-responsive genes depended upon BPTF, suggesting that NURF functions as a transcription cofactor for SMAD [87]. SNF2L is also critical for granulosa cell proliferation and differentiation during folliculogenesis [88, 89]. *Snf2l* mutant mice responded differently under gonadotropin induction, and thus, they yielded significantly fewer eggs and exhibited fewer secondary follicles compared to control WT mice. The study also indicated that *Fgl2* transcription, which can encode a prothrombinase for mouse reproduction to mediate folliculogenesis, is regulated by *Snf2l* [89].

Many genes are heterochromatinized upon differentiation, and thus, regularly spaced nucleosomes are needed for higher order compaction. The ISWI-containing chromatin remodeling complex ACF1 is required for nucleosome assembly. In the meantime, centromeric chromatin is assembled by RSF1, while heterochromatin formation is regulated by NoRC; thus, rDNA repeats can be silenced [90–92]. Deficiency in either *Drosophila* ISWI or BPTF leads to repressed histone H1 levels and a general male X chromosome decondensation [93, 94]. Therefore, ISWI-regulated histone H1 deposition and nucleosome spacing result in higher order chromatin structures and gene repression, which play an important role during the transition between the progenitor cell and the differentiated cell fate [95].

Taken together, ISWI complexes have been shown to have specific roles in cell proliferation, differentiation, or maturation (Table 1). SNF2H-containing ISWI complexes mainly participate in early development and progenitor cell proliferation, while the complexes containing SNF2L are mostly involved in cell differentiation and maturation.

### 3.3. CHD

Nine chromodomain helicase DNA-binding (CHD) proteins (CHD1-9), which can either function alone or cooperate with other proteins to form the complexes, constitute a CHD subfamily. Among them, different CHD complexes have distinct roles in early development and cell lineage differentiation.

CHD1 has been shown to be required for maintaining the self-renewal ability and pluripotency of embryonic stem cells [96, 97]. CHD1 was found to interact with RNA polymerases I/II to regulate the transcription of both rRNA and mRNA and maintain proper transcriptional output [98]. CHD1 is also involved in endothelial to hematopoietic transition (EHT), by which hematopoietic stem cells and progenitors derive from endothelial cells in various organs. However, CHD1 is not essential before or after hematopoietic stem cell and progenitor formation, and CHD1 functions to induce the high transcriptional output of hematopoietic progenitors only in a specific time window [99].

In the developing brain, the NuRD complex, which contains CHD4, is required for synapse formation [100]. This complex can compromise a set of developmentally downregulated genes in presynaptic granule neurons to drive synaptogenesis. However, CHD5 is involved in neuronal differentiation to inhibit nonneuronal lineage genes [101–103]. In addition, CHD7 is essential for maintaining the quiescence of neural stem cells in adults by repressing a number of cell cycle activators and inducing Notch signaling [104]. Moreover, CHD7 is critical for neurogenesis during the morphogenesis of the inner ear [105]. In contrast, CHD8 is associated with autism spectrum disorder (ASD). By decreasing half the dose of *Chd8* in neural progenitor cells, the neural developmental genes containing those ASD-related genes were downregulated [106].

CHD complexes also play an important role in heart development. A NuRD complex containing CHD3 or CHD4 is involved in the proliferation of cardiomyocytes by interacting with the transcription factor FOG2 [107]. Once the interaction between FOG2 and NuRD is impaired, it may lead to perinatal lethality because of a thin ventricular myocardium and defects in the atrial and ventricular septum. The FOG2-NuRD interaction maintains cardiomyocyte proliferation by inhibiting *Cdkn1a*, which is a cell cycle inhibitor gene. Therefore, the disruption phenotype in the *FOG2-NuRD* interaction can be rescued through *Cdkn1a* deletion. Furthermore, CHD7 is involved in transcription activity in various heart development processes. *Chd7* mutant mice exhibited CHARGE syndrome in cardiac aspects [108, 109] while CHD7 mutations have been discovered in sporadic cases in congenital human heart defects [110].

In a well-established MSC model with the induction of osteoblast lineage differentiation, CHD1 is essential for osteogenesis by regulating the transcriptional program of osteoblast differentiation, specifically at later stages. Moreover, CHD1 depletion was shown to reduce the induction of lineage-specific genes in adipocyte differentiation, indicating that CHD1 has a more general role in regulating transcriptional programs related to MSC differentiation [111]. CHD7 is also important in osteogenic differentiation since the expression of CHD7 can be induced in MSCs under osteogenic induction medium conditions while CHD7 depletion in MSCs leads to the repression of several osteogenic transcription factors and decreased MSC osteogenesis capability [112]. ChIP analysis showed that CHD9 can bind to skeletal tissue-specific promoters expressed at different stages during osteoprogenitor differentiation. The interactions between CHD9 and the promoter regions involved in the osteogenic process demonstrate the importance of CHD9 in the transcription process in osteoprogenitor cells and its possible role in the MSC maturation direction [113–115]. Another study indicated that nucleolar CHD9 acts as a ribosomal gene transcription regulator, which has also been implicated in cell fate and differentiation of MSCs [116].

Overall, CHD complexes function to regulate transcription or suppression of different genes and induce various lineage differentiations in MSCs (Table 1). This process relies on the cooperation of CHD complexes with histone modifiers and transcription factors specific to different lineages.

### 3.4. INO80/SWR

The ATPase subunits of INO80/SWR are another subfamily of ATP-dependent chromatin remodeling complexes that exhibit a conserved insertion in the ATPase/helicase domain. This is required for the interaction between RVB1/RVB2 helicase and these complexes [117]. The INO80 subfamily includes the INO80 complex [118], while SWR is comprised of P400/TIP60 and SRCAP [119]. Histone variant H2A.Z exchange and ATP-dependent nucleosome mobilization are present in INO80-involving chromatin remodeling [120]. However, SWR complexes are mostly required in the process of H2A.Z deposition into nucleosomes that contain H2A [117].

MSCs transfected with siRNAs targeting INO80 resulted in an impaired mineral deposition in osteogenic induction conditions, and the implanted mice with INO80-silencing MSCs also exhibited decreased bone formation. This suggests the essential role of the INO80 complex in MSC osteogenic differentiation and its potential application in tissue engineering in the clinic and osteoporosis treatment [121] (Table 1). INO80 is critical for meiotic recombination during spermatogenesis [122]. A conditional *Ino80* mutation in spermatogonia before meiosis led to reduced synapse formation and double-strand break defects [123, 124]. P400 (EP400), the subunit of the SWR complex, plays an important role during hematopoiesis by regulating the expression of several embryonic globin genes and deregulating HOX gene expression [125, 126]. In bone marrow cells, P400 conditional knockout led to impaired stem and progenitor cell pool of hematopoiesis because of the progression defects in the cell cycle [125]. Moreover, P18^Hamlet^ (ZNHIT1), a SRCAP subunit, is required for muscle differentiation [127]. P18^Hamlet^ is phosphorylated at the promoter region of *Myog*, a muscle-specific transcription factor gene. H2A.Z is then recruited to phosphorylated P18^Hamlet^/SRCAP, forming the chromatin structure necessary for *Myog* transcription.

## 4. Conclusion

Over the past years, MSCs have become the focus of intense interest. Thus, they have been investigated for their capacities for self-renewal and lineage specification. The application of MSCs has been considered as a solution for the poor ability of adult tissue regeneration and a potential treatment for human diseases. Gene expression programs work at the chromatin level, so the organization of chromatin is essential in both normal and malignant development and tissue regeneration. We propose that the efficiency of differentiation of MSCs into a variety of cell types will be enhanced by modifying the composition of ATP-dependent chromatin remodeling complexes. As mentioned above, ATP-dependent chromatin remodeling complexes catalyze critical functions in self-renewal and multilineage differentiation of MSCs. In addition, the role of ATP-dependent chromatin remodeling in embryonic stem cells in diverse tissue types also raises the possibility that it may have similar functions in MSCs. The diversity of combinations of multiple subunits has specific functions in chromatin remodelers. For example, a specific combination plays an important role in ATP-dependent chromatin remodeling in differentiated cells, while another combination is crucial for some tissue progenitors. Moreover, ATP-dependent chromatin remodelers can regulate specific transcription in various cell types or with different transcriptional programs in the same cell type depending on the collaboration of these chromatin remodelers with histone-modifying complexes that can induce the binding of histone marks to regulatory sites. The insights of the ATP-dependent chromatin remodeling complexes and their roles in MSC fate determination will provide potential strategies for regeneration and cell-based tissue-engineering therapy.

## Figures and Tables

**Table 1:** Types of ATP-dependent chromatin remodeling complexes and their subunits in different lineage specification.

Lineage	Types of ATP-dependent chromatin remodeling complexes			
	SWI/SNF	ISWI	CHD	INO80/SWR
Osteogenesis	BRG1, BAF47, BAF200, BAF180, BRD7		CHD1, CHD7, CHD9	INO80
Neurogenesis	BRG1, BAF45A, BAF53A, BAF53B	SNF2H, SNF2L	CHD4, CHD5, CHD7, CHD8	
Adipogenesis	BRG1, BRM, BAF47			
Cardiomyocytes	BRG1, BAF250A, BAF60C		CHD3, CHD4, CHD7	
Hematopoiesis	BAF180, BAF45A	SNF2H, SNF2L	CHD1	P400
Hepatocytes	BRG1, BRM, BAF250A, BAF47, BAF60A			
Chondrogenesis	BRG1			
Muscle cells				ZNHIT1
